# An unusual cause of chest pain:case report

**DOI:** 10.1186/1477-7800-4-11

**Published:** 2007-05-03

**Authors:** Ashok Subramanian, Hilary Birch, Adam Stacey-Clear

**Affiliations:** 1 Department of General Surgery, East Surrey Hospital, Canada Avenue, Redhill, Surrey, UK; 2 Department of Histopathology, East Surrey Hospital, Canada Avenue, Redhill, Surrey, UK

## Abstract

**Background:**

Sarcomas form a heterogenous group of relatively uncommon malignant tumours which are derived from connective tissue components. In total they comprise approximately 1% of all new cancers diagnosed per year in the United Kingdom (UK). As subset of this, the 'Unclassified' Sarcoma forms approximately 4% of the total [1]. They often present with as relatively slow growing, asymptomatic masses and as such may often be misdiagnosed as in this case.

**Case presentation:**

A 52 year old man presented to his general practitioner (GP) with left sided chest pain. A strong family history of ischaemic heart disease prompted hospital referral and further investigations which all proved negative for coronary artery disease. Following weight loss and ongoing chest pain, he represented to his GP with a hard mass arising from the left pectoralis major muscle at the site of the previous pain. Surgical excision followed by later compartectomy revealed an unclassified low grade Sarcoma with lymphoma like features.

**Conclusion:**

In this case, chest pain masquerading as ischaemia, may have been caused by peri-neural infiltration or compression of adjacent muscle bulk by tumour, with eventual surgical resection providing a good long term prognosis.

## Case report

### Background

Sarcomas form a heterogenous group of relatively uncommon malignant tumours which are derived from connective tissue components. In total they comprise approximately 1% of all new cancers diagnosed per year in the United Kingdom (UK). As subset of this, the 'Unclassified' Sarcoma forms approximately 4% of the total [1]. They often present with as relatively slow growing, asymptomatic masses and as such may often be misdiagnosed as in this case.

### Case presentation

A 52 year old Caucasian gentleman presented to his general practitioner (G.P) with moderate left sided chest pain. This pain had developed suddenly that morning and was localised to the chest. Due to a strong family history of ischemic heart disease and the nature of the pain, he was then referred to the local casualty department.

All investigations including chest radiographs, electrocardiograms, cardiac enzymes, echocardiograpy and subsequent coronary angiography proved negative for coronary artery disease. He was advised by his hospital practitioner that he may have had a chest infection, and as part of a general lifestyle change was advised to try to take more exercise and lose weight. Over the next four months Mr DP succeeded in losing 20 kg, but still complained of worstening left sided chest pain. This pain was not affected by exercise, movement or heavy lifting.

He then re-presented to his G.P with a hard mass in the left upper chest over the site of his longstanding chest pain. He was referred for a surgical consultation and underwent excision of a lesion arising from the left pectoralis major muscle 1 week later.

**Figure 1:**
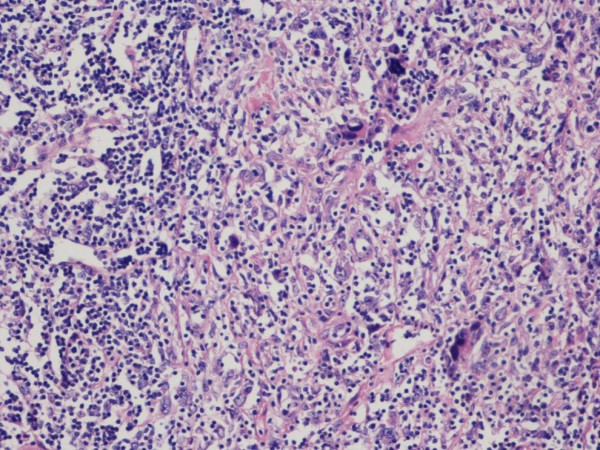
Histological section of intramuscular tumour showing bizarre malignant cells (arrowed) and adjacent lymphoid population H&E ×400

Histology from this lesion, revealed features suggestive of Hodgkin's Lymphoma, but due to the unusual appearance of the lesion the biopsies were sent for a second opinion. A second opinion noted mixed chronic inflammatory cells with occasional germinal centres which might suggest an inflammatory pseudotumour. The ususual features of very large bizarre, and atypical polygonal cells however led to a final diagnosis of an unclassified sarcoma, morphologically low grade, with lymphoma like features (Fig 1). Personal communication from Professor Fletcher recommended annual clinical follow-up with annual MRI scanning.

Of note, Mr DP's chest pain completely disappeared following excision of his lesion.

Annual scans were clear of recurrent local disease but Mr DP represented in November 2005 with a nodule adjacent to the scar from previous surgery. Excision of this lesion revealed recurrent tumour of the same morphology and he underwent a radical compartectomy at a third operation in December 2005.

## Conclusion

Sarcomas form a heterogenous group of relatively uncommon malignant tumours which are derived from connective tissue components. In total they comprise approximately 1% of all new cancers diagnosed per year in the United Kingdom (UK). As subset of this, the 'Unclassified' Sarcoma forms approximately 4% of the total^1 ^and may often be misdiagnosed.

The majority of patients present with asymptomatic, deep seated, slow growing, ill defined masses. In this case, the chest pain may have been due to perineural invasion and expansion of the tumour with compression of the surrounding muscle. It became palpable as a result of intentional weight loss and increasing size of the tumour.

Tumours < 5 cm (as in this case) can be effectively treated with wide local excision ensuring margins of at least 2 cm, with 5 year disease free survival rates of nearly 78% in low grade lesions [2] Larger more aggressive tumours or recurrent tumours may benefit from more radical surgery or radiotherapy.
